# In Vitro Investigation of the PneumoWave Biosensor for the Identification of Central Sleep Apnea in Pediatrics

**DOI:** 10.3390/bios16020077

**Published:** 2026-01-27

**Authors:** Burcu Kolukisa Birgec, Ross Langley, Jennifer Miller, Osian Meredith, Beyza Toprak, Alexander Balfour Mullen

**Affiliations:** 1 Strathclyde Institute of Pharmacy and Biomedical Sciences, University of Strathclyde, Glasgow G4 0RE, UK; burcu.kolukisa@strath.ac.uk (B.K.B.); beyza.toprak.2022@uni.strath.ac.uk (B.T.); 2 Royal Hospital for Children, Glasgow G51 4TF, UK; ross.langley@ggc.scot.nhs.uk; 3 PneumoWave Ltd., Motherwell ML1 4WQ, UK; jennifer.miller@pneumowave.com (J.M.); osian@pneumowave.com (O.M.)

**Keywords:** central sleep apnea, infancy, apnea monitor, manikin simulation, biosensor

## Abstract

The interpretation and diagnosis of central sleep apnea in pediatrics by nocturnal polysomnography is challenging due to its technical complexity, which involves the simultaneous recording of multiple physiological parameters related to sleep and wakefulness. Furthermore, the unfamiliar environment of a sleep laboratory can hinder sleep evaluation, and diagnostic backlogs are common due to restricted capacity at specialist tertiary centers. The ability to undertake home sleep studies in a familiar environment using simple, robust, and low-cost technology is attractive. The potential to repurpose the PneumoWave biosensor, a UKCA Class 1 device, registered as an accelerometer-based monitoring device that is intended to capture and store chest motion data continuously over a period of time for retrospective analysis, was explored in an in vitro model of central sleep apnea. The PneumoWave system contains a biosensor (PW010), which was able to record simulated apnea episodes of 5 to 20 s across physiologically relevant pediatric breathing rates using an in vitro manikin model and manual annotation. The findings confirm that the PneumoWave biosensor could be a useful technology to support home sleep apnea testing and warrant further exploration.

## 1. Introduction

The American Academy of Sleep Medicine (AASM) defines central sleep apnea (CSA) in pediatric patients as a complete absence of inspiratory effort throughout its duration, and satisfies at least one of the following conditions: the event lasts ≥20 s, the event is associated with either an arousal, an awakening or a ≥3% drop in oxygen saturation or for infants under 1 year of age, the event is accompanied by bradycardia, (a heart rate < 50 beaths per minute) for ≥5 s or <60 bpm for ≥15 s [1]. One study retrospectively analyzing the polysomnography of 2981 children with a central sleep apnea index of five or more events per hour indicated that 3% presented central sleep apnea, with Chiari malformation being the most prevalent cause [2]. The consequences of central sleep apnea are not as thoroughly investigated as those of obstructive sleep apnea [3]. However, central sleep apnea activates the sympathetic nervous system, causes oxidative stress and systemic inflammation in addition to impacting upon heart rate and blood pressure [3,4].

The early detection of central sleep apnea is crucial to prevent or minimize potential complications associated with the condition. Polysomnography is recognized as the diagnostic gold standard [5]. Polysomnography simultaneously captures an array of physiological parameters related to sleep and wakefulness, including electroencephalogram (EEG), electrocardiogram (EKG), electromyogram (EMG), airflow, blood oxygen saturation, and chest movements [6,7,8]. The complexity of polysomnography can result in pediatric distress and lead to disrupted, atypical sleep patterns, which hinder the interpretation and diagnosis of collected data [8,9]. Execution of polysomnography requires regular adjustment of equipment over the data collection period, which imposes an additional burden on staff [10,11,12]. Collected data then requires retrospective manual scoring by a respiratory physiologist following AASM guidelines. Furthermore, diagnostic backlogs are common due to capacity issues accessing clinical services at specialist tertiary centers [13].

In recent years, advancements in devices, particularly wearable technology, have gained considerable attention. These wearable devices can assist parents and healthcare providers in prospectively monitoring children in a home environment. Commercial monitors in the market to detect cessation of breathing or movement of pediatrics, include Nanny Baby, Snuza HeroMD, SISS Babycontrol, and Nanit, but only a select few have a regulatory medical device classification [14,15,16,17]. While medical-grade alarms such as the Snuza HeroMD or SISS Babycontrol^®^H provide essential safety alerts, they typically function as ‘black-box’ systems, restricting user/healthcare professional access to the underlying physiological data. Similarly, consumer wellness monitors (e.g., Nanit) focus on sleep trends and video monitoring rather than diagnostic precision. A key differentiator of the PneumoWave system, as highlighted in Table 1, is the accessibility of full raw respiratory waveforms combined with a wireless, wearable device. Unlike wired electrodes (e.g., SISS) that restrict mobility, or consumer devices that offer only summary metrics, our system enables detailed retrospective analysis of breathing patterns. This capability is critical for clinical research and the precise diagnosis of central sleep apnea, moving beyond simple event detection to detailed physiological characterization. Also, these devices mostly detect 10 s breath cessations and/or no movement for 20 s, and some provide gentle vibrations to rouse pediatrics and/or give alerts to guardians. However, these devices have limitations, including their dependence on wired connections and their inability to record respiratory patterns [18]. An unintended consequence of these devices may be stress and anxiety experienced by parents due to frequent false alarms, which can detrimentally impact their quality of life [19,20]. These limitations emphasize the need for innovative medical devices that offer advanced capabilities for accurate, non-invasive, and continuous monitoring of breathing patterns in home settings.

PneumoWave biosensor, a registered UKCA Class 1 medical device, is intended to capture and store chest motion data continuously over a period of time for retrospective analysis. Its 3-axis accelerometer biosensor allows for recording precise chest motion when mounted on the chest. This study aims to investigate the performance of the PneumoWave biosensor in recording simulated CSA using an in vitro manikin model and manual annotation of the device output.

## 2. Materials and Methods

### 2.1. Hardware and Software Configuration

The PneumoWave biosensor used in this study was previously described in detail by Gonzalez Utrilla et al. (2025), who reported the device’s technical development and validation framework for central body motion monitoring [21]. The PneumoWave, PW010(PneumoWave, Maxim Park, UK), is a non-invasive biosensor with a diameter, height, and weight of 42 mm, 18 mm, and 22 g, respectively. It is powered by a replaceable CR2032 battery, providing it with a 4-day operational life for continuous monitoring. It is used to measure chest wall movement and has a measurement range of ±2 g, an accuracy of ±0.05 g, and a sensitivity of less than 0.005 g, making it suitable for capturing subtle respiratory motions against gravity. The biosensor attaches to the skin via a user-friendly adhesive EKG electrode, which can be easily replaced. Data is exported from the biosensor via Bluetooth and captured on a PneumoWave biosensor app (version 1.0.3) operated on a Samsung Galaxy A7 Lite tablet (LTE model, Android 11, Samsung, Suwon, South Korea)

**Figure 1:**
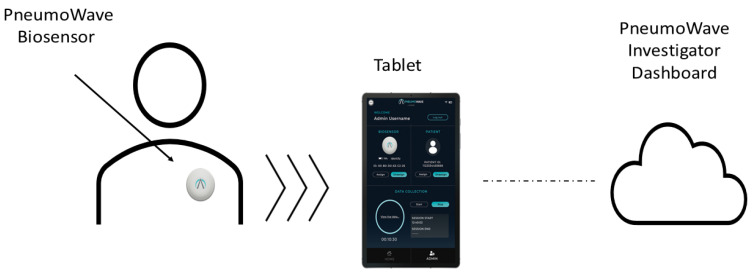
Hardware and software configuration for recording chest motion using the PneumoWave biosensor integrated platform of patient data capture.

An integrated green light indicator confirms when the device is connected to the app. The PneumoWave biosensor app serves to streamline data via Wi-Fi to a storage cloud from where it can be viewed using the PneumoWave Investigator Dashboard (PneumoWave, Maxim Park, UK; Figure 1).

### 2.2. In Vitro Manikin Apnea Model

#### 2.2.1. Preliminary Short-Interval Measurements Using the PneumoWave Biosensor

**Figure 2:**
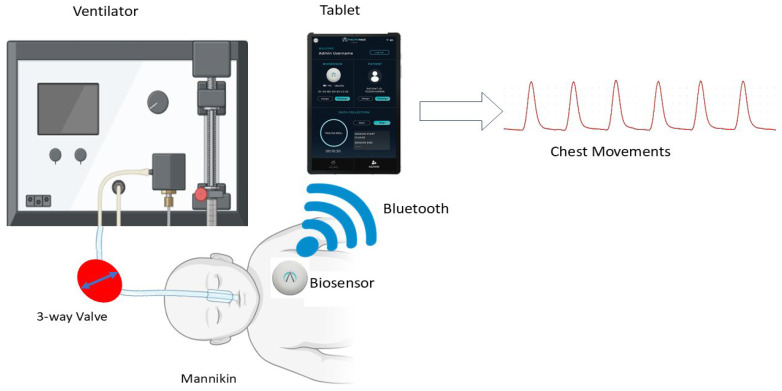
An in vitro manikin model to investigate using the PneumoWave biosensor to record motion with a simulated sleep apnea model.

A PractiBaby Infant CPR manikin (PractiMan, Colchester, UK) with an external 50–60 mL lung bag (SP-001(PB), PractiMan, UK) or a SimJunior manikin (Laerdal Medical, London, UK) with an internal 150–205 mL lung bag was connected to an LTV 1150 ventilator (Vyaire Medical, Basingstoke, UK: operational tolerance of ±1 breath per minute) via an endotracheal tube (Portex Tracheal Tube 4.0 mm ID cuffed, AHP Medicals, Cardiff, UK). For the PractiBaby manikin, an inline 3-way valve (B08B4M38X5, WGD, Shengli Coalfield, China) was used, whereas the SimJunior manikin was ventilated without the 3-way valve due to the internal lung system design. The PneumoWave biosensor was positioned on the upper left region of each manikin’s chest, approximately 6 cm below the center of the left collarbone, and mounted to an EKG electrode (Figure 2).

Each data collection phase included 5 replicates of 10 min each, which allowed simulated respiratory cycle measurements between the range of 6 to 80 breaths per minute (BPM). During each measurement, the ventilator was operated at the lowest available setting that allowed visible chest movements on the manikin. During apnea simulations, either the 3-way valve was manually closed, or the tube was manually disconnected to immediately prevent air ingress into the artificial lungs for periods lasting between 5 and 20 s before recommencing a normal respiratory pattern for 1 min before repeating a simulated apnea an additional 4 times.

#### 2.2.2. Long-Interval Measurements Using the PneumoWave Biosensor

To better simulate real-life clinical scenarios, an alternative manikin system was used to generate dynamic physiological changes seen in young children. In these experiments, a Resusci Baby manikin with internal lung system (Laerdal Medical, UK), was connected to a SLE6000 ventilator (Inspiration Healthcare, Croydon, UK: operational tolerance of ±1 breath per minute until 70 BPM, ±2 breath per minute at 71–120 BPM) via an endotracheal tube (Portex Tracheal Tube 4.0 mm ID, AHP Medicals, UK) without 3-way valve. The biosensor was positioned in the same way as short-interval measurements. A dynamic breathing range was generated using a random number generator to assign a respiratory rate between 6 and 80 breaths per minute (www.random.org, accessed on 23 May 2024). Individual breathing rates were held for a period of 10 min before alteration. Periods of apnea (5–20 s) were simulated as described in the short-interval protocol by physically disconnecting/reconnecting the endotracheal tube. Each 5 h experiment was repeated five times. Experimental conditions are summarized in Table 2.

### 2.3. Data Analysis

Raw data captured using the PneumoWave biosensor was exported to the PneumoWave Investigator Dashboard and downloaded as a CSV file. The CSV file was imported into MATLAB (R2023a, MathWorks, Inc., Natick, MA, USA) for visualization and analysis. When the manikin was used in the supine position, the biosensor accelerometer had identical vectors in the x, y, and z-planes. For this reason, only the y-plane was used for measurement determination.

Manual Average Visual Count (MAVC) was calculated for each data set. Manual Average Visual Count was obtained by manually counting the total number of whole simulated breaths per minute. The obtained MAVC was compared to individual ventilator settings using a linear regression and correlation model to understand the associated fit using a 95% confidence interval. Bland–Altman plots using a 95% confidence interval (CI) limits of agreement (LoA) were also performed on MAVC and ventilator setting data sets to identify whether any bias between the mean differences was due to fixed or random events.

**Figure 3:**
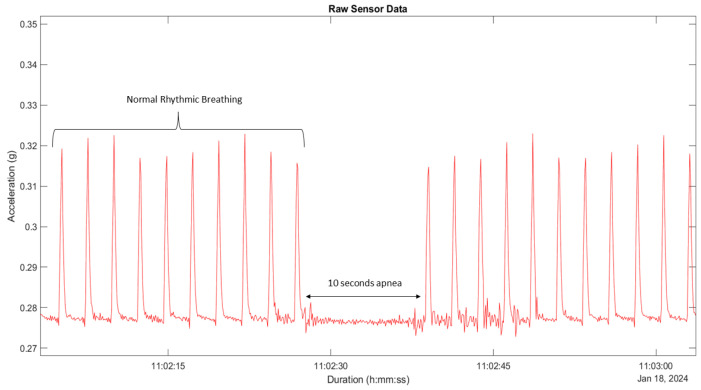
Showing normal rhythmic breathing and a 10 s simulated apnea event at a 25 BPM setting.

Simulated apnea durations were defined as the time interval (in seconds), predetermined by the researcher to range between 5 and 20 s, during which ventilator airflow was intentionally interrupted to produce controlled apnea events. These intervals were then used to identify the corresponding absence of simulated chest movements in biosensor data. In the simulated apnea analysis, apneic pauses were identified in MATLAB as periods where the accelerometer amplitude remained <0.002 g for longer than two consecutive respiratory cycles. This threshold was empirically determined from baseline noise observed during apnea segments across all manikin models (Figure 3). This value represents a conservative threshold positioned effectively above the sensor’s intrinsic noise and below the lowest amplitude of respiratory motions. This empirical finding is consistent with the device’s technical validation framework recently published by Gonzalez Utrilla et al. (2025) [21], which characterizes the sensor’s activity thresholds and demonstrates clear signal differentiation between physiological motion and instrument noise. Means and standard deviations were calculated for each apneic event. To evaluate differences in the use of a valve or manual disconnection across manikin models, Levene’s test was used to assess the homogeneity of variances. If inequality was observed, Welch’s ANOVA was applied to the data. All statistical analyses were conducted at a significant level of *p* < 0.05.

## 3. Results

### 3.1. Normal Rhythmic Breathing

**Figure 4:**
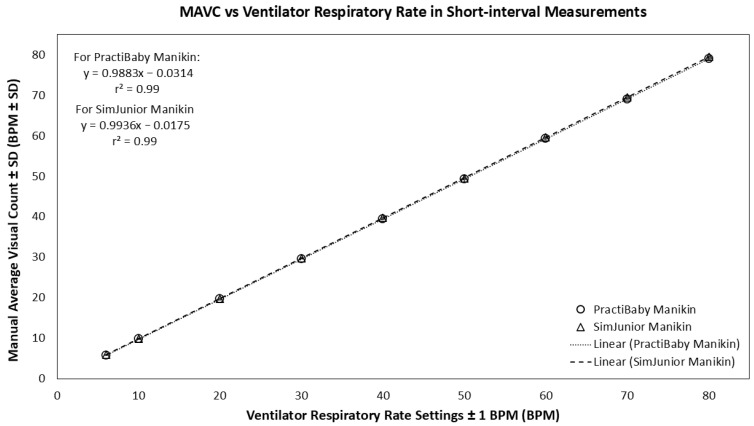
The scatterplot comparing MAVC from the PneumoWave device versus the Ventilator Respiratory Rate with standard deviation (n = 5) in short-interval measurements. Circles represent the PractiBaby manikin model, and triangles represent the SimJunior manikin model. Error bars representing standard deviation are included but may be barely visible due to the high reproducibility and low variance of the mechanical simulation data.

In the short-interval measurement, Manual Average Visual Count (MAVC) of the PneumoWave biosensor raw data accurately measured normal rhythmic simulated breathing with a strong positive linear correlation (r^2^ value of 0.99; 95% confidence interval, CI = 0.99–1.00, *p* < 0.05) between MAVC and ventilator respiratory rates observed, with a standard deviation range of 0 to 0.27 in the PractiBaby model. For the SimJunior model, a similar linear correlation (r^2^ value of 0.99; 95% confidence interval, CI = 0.99–1.00, *p* < 0.001) was observed between MAVC and ventilator respiratory rates, with a range of standard deviation 0.07 to 0.80 (Figure 4).

**Figure 5:**
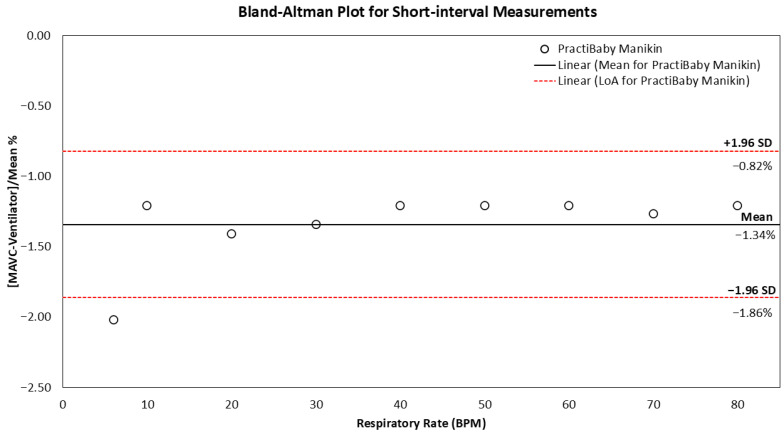
The Bland–Altman plot of the difference between the MAVC and ventilator respiration rate settings across the 6–80 BPM range for short-interval measurements on the PractiBaby manikin model. The red dotted horizontal lines show the upper and lower 95% limits of agreement. The black horizontal line shows the mean difference between MAVC and ventilator settings.

As illustrated in Figure 5, the Bland–Altman plot provides a comprehensive representation of agreement metrics between MAVC data recorded using the PneumoWave device and the ventilator in the PractiBaby manikin. The analysis reveals that the lower limit of agreement was −1.86%, while the upper limit was −0.82%. The calculated mean difference among the measurements was −1.34%. Excursions outside the 95% CI of LoA were observed for 6 BPM (−2.02%). Similar results were observed for the SimJunior model. The lower LoA was −1.16%, the upper limit was −0.18%, and the mean difference was −0.67%. No data falls outside the limits of agreement.

**Figure 6:**
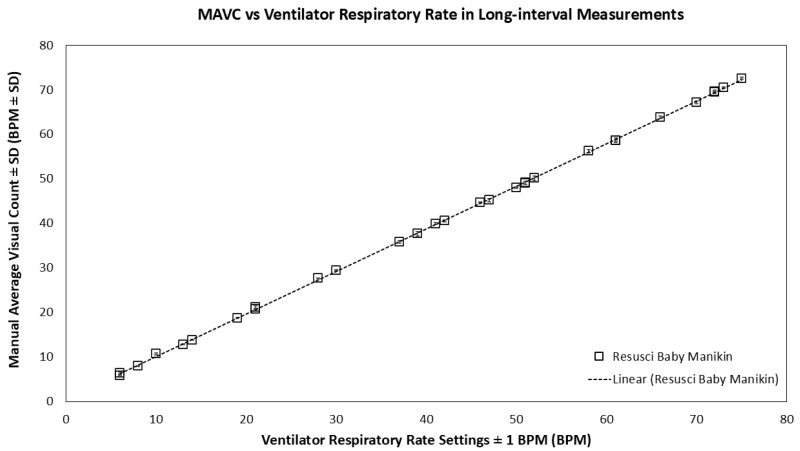
Manual Average Visual Count (±SD) from PneumoWave device during 5 h recording with the dynamic respiratory range between 6 and 80 BPM in Resusci Baby manikin model (n = 5).

**Figure 7:**
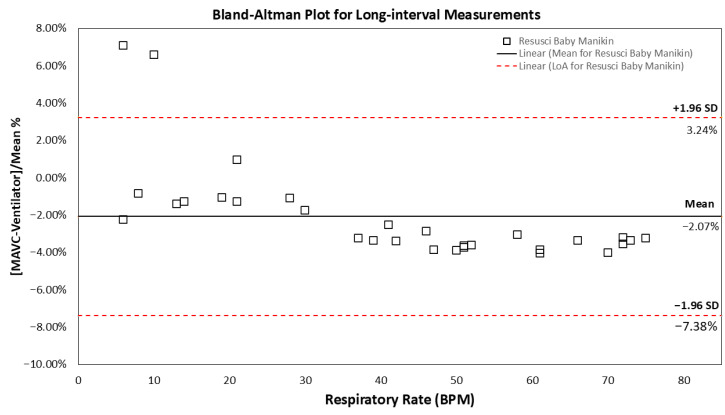
The Bland–Altman plot of the difference between the MAVC against the ventilator respiration rate of random dynamic ranges between 6 and 80 RPM (n = 5) in the long-interval measurements. The dotted horizontal red lines show the upper and lower 95% limits of agreement. The black horizontal line shows the mean of the difference between MAVC and ventilator settings in the Resusci Baby manikin model.

During long-interval measurements, a similarly high correlation between MAVC and ventilator respiratory rate settings was observed (Figure 6: r^2^ value of 0.99; 95% confidence interval, CI = 0.99–1.00, *p* < 0.05; standard deviation ±0.05–0.55). As illustrated in Figure 7, the Bland–Altman analysis reveals that the mean and 95% CI of LoA values were −2.07%, −7.38%, and 3.24%, respectively. Excursions outside the 95% CI were observed at 6 BPM (7.07%) and 10 BPM (6.58%), and a slight negative trend was noted at higher respiratory rates.

### 3.2. Simulated Apneas

**Figure 8:**
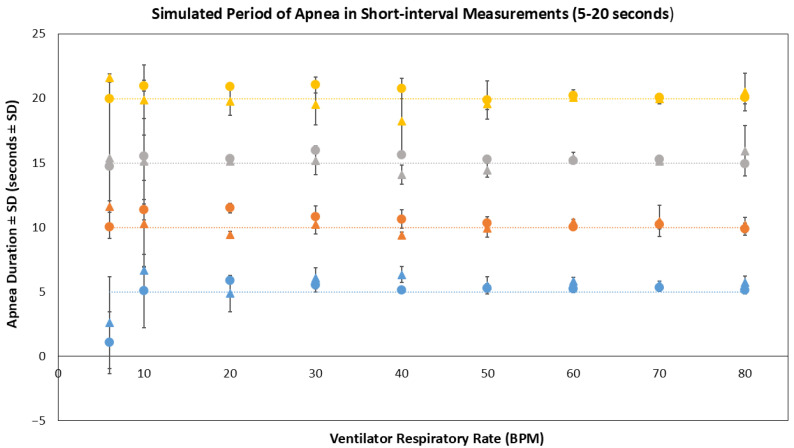
Simulated periods of apnea ranging from 5 to 20 s whilst operating the manikins at 6 to 80 BPM during 10 min in vitro data collection (n = 5). Circles represent data collected using the PractiBaby manikin model, whilst triangles represent data collected using the SimJunior manikin. Blue data points represent 5 s periods of apnea; orange data points represent 10 s periods of apnea; gray data points represent 15 s periods of apnea, and yellow data points represent 20 s periods of apnea.

In the short interval experiments, simulated periods of apnea ranging from 5 to 20 s were successfully identified across the range of 10 to 80 BPM at time points predetermined by the researcher. However, at the lowest setting of 6 BPM, when the interval between two consecutive breaths is 10 s, shorter periods of simulated apnea could not be detected if they occurred between consecutive breaths (Figure 8). However, the biosensor successfully recorded normal rhythmic motion, interpreted by the MAVC method to be simulated breathing before/after the apnea episode of varying durations—these apnea episodes were measured using manual annotation. The mean duration (±SD) of 5 s, 10 s, 15 s, and 20 s apnea events in the PractiBaby manikin model recorded were 4.86 ± 0.82 s, 10.54 ± 0.47 s, 15.33 ± 0.03 s, and 20.44 ± 0.54 s, respectively. The mean duration in the SimJunior manikin model was 5.47 ± 0.93 s, 10.22 ± 0.85 s, 15.10 ± 1.65 s, and 19.90 ± 1.17 s, respectively.

**Figure 9:**
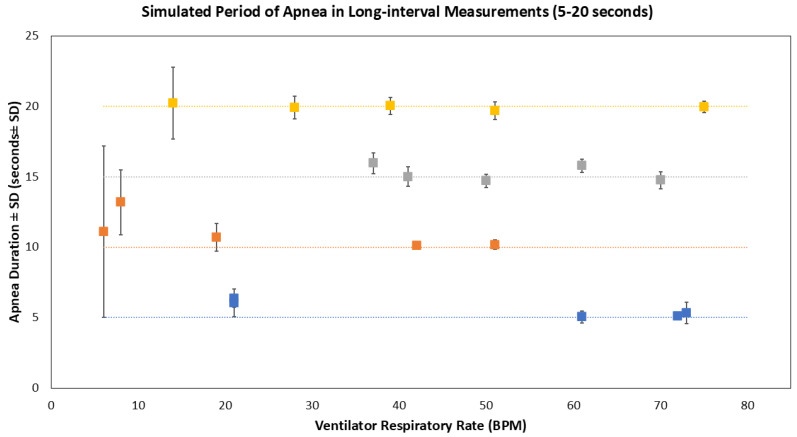
Observed 5-, 10-, 15-, and 20 s simulated apnea events in each experiment in the Resusci Baby manikin model (n = 5). Blue data values represent the mean duration of 5 s apnea per BPM, orange for 10 s, gray for 15 s, and yellow for 20 s. Control lines correspond to these apnea durations. Blue data points represent 5 s periods of apnea; orange data points represent 10 s periods of apnea; gray data points represent 15 s periods of apnea, and yellow data points represent 20 s periods of apnea.

In long-interval experiments on the Resusci Baby manikin, all 5-, 10-, 15-, and 20 s apnea events were observed. The mean duration (±SD) of 5 s, 10 s, 15 s, and 20 s apnea events recorded were 5.59 ± 0.57 s, 11.05 ± 1.99 s, 15.24 ± 0.60 s, and 19.96 ± 0.99 s, respectively (Figure 9). Analysis using Welch’s ANOVA test indicated no significant differences (*p* > 0.05), indicating that the use of a valve or manual disconnections during periods of simulated sleep apnea provided similar performances.

## 4. Discussion

The results of this in vitro study demonstrated the potential of the PneumoWave biosensor as a non-invasive, accurate, and efficient tool for recording chest motion in pediatrics. The biosensor successfully recorded simulated respiratory patterns that strongly correlated with ventilator settings, as evidenced by the Bland–Altman analysis. Importantly, the PneumoWave biosensor was effective in recording simulated apnea events at predetermined time points, particularly in the longer duration dynamic respiratory rate simulations. Although the ventilator is not a clinical method for measuring respiratory rate, the Bland–Altman plot shows strong agreement. A slight negative trend was observed, suggesting that the difference between the biosensor and ventilator measurements increases with respiratory rate. Ventilators can introduce errors or approximations into respiratory rate measurement, particularly at higher respiratory rate values [28,29]. The ventilator self-calibrates during transition from high to low respiratory rates within long-interval dynamic range measurements. The ventilator’s automated correction procedure may explain the small differences in values between the ventilator and the biosensor during long-interval measurements. However, good overall concordance was observed.

The in vitro models were a particularly stringent and challenging test of the hardware’s performance. The performance evaluation of the PneumoWave biosensor showed that it successfully captured short apneic events of 5, 10, and 15 s with greater sensitivity than that required by the AASM CSA criteria.

The PneumoWave biosensor used in this study was previously described in detail by Gonzalez Utrilla et al. (2025), who reported the device’s technical development and validation framework for central body motion monitoring [21]. The present study evaluated the biosensor’s performance in detecting simulated apnea events in a controlled in vitro environment in pediatric settings. While this in vitro study utilized static manikins to validate signal fidelity, clinical environments introduce challenges, such as motion artifacts and sensor displacements. The PneumoWave biosensor systems incorporate motion-rejection algorithms for adult populations [21], and the suitability of these and pediatric-specific algorithms will be determined in human volunteer studies in due course.

The use of a PneumoWave biosensor could offer several advantages over polysomnography and commercially available devices in a pediatric setting, with its portability enabling patients to undergo screening in a more comfortable home environment. Furthermore, the transition from hospital to home care requires robust remote monitoring solutions that go beyond simple data logging. Recent advancements have demonstrated the feasibility of IoT-enabled Non-Invasive Ventilation (NIV) devices that transmit real-time flow and pressure data to the cloud, as described by Menniti et al. 2024 [30]. In this context, the PneumoWave biosensor can be designed to function as a complementary node within such a connected ecosystem. While the NIV system monitors the therapy delivery (air pressure and flow), the PneumoWave system provides independent verification of the physiological response (chest wall mechanics). By integrating these two data streams—machine output versus patient effort—clinicians could remotely distinguish between effective ventilation and machine-delivered breaths that fail to produce adequate chest excursion. Therefore, the proposed sensor validates the ‘co-design’ strategy advocated in the recent literature, acting not as a standalone device but as a critical component of a multi-modal home monitoring suite.

The biosensor was designed for user-friendliness and cost-effectiveness, providing basic output that makes it readily accessible for both patients and healthcare providers. The introduction of downstream processing to replace the manual annotation could assist healthcare professionals in making more informed decisions about diagnosis and treatment. Simple integration with a portable pulse oximeter to capture oxygen saturation and utilization of an additional biosensor on the abdomen could allow confirmation of diaphragmatic breathing, common in pediatrics, and enhance diagnostic potential. The present findings complement earlier projects, such as the OD-SEEN study and subsequent developments of the PneumoWave DCM and ALERT systems [21], by providing preclinical evidence supporting the sensor’s accuracy in potential respiratory event detection.

While mechanical simulations provide a standardized environment for sensor validation, they cannot fully replicate the complex, spontaneous movements and skin-to-sensor coupling variations seen in clinical environments. However, the use of both terms (PractiBaby and Resusci) manikins confirms the sensor’s adaptability to varying anatomical body designs. Furthermore, the high fidelity of the accelerometer data suggests that distinguishing between rhythmic respiratory efforts and irregular, high-amplitude body movements is feasible. This distinction is supported by the device’s established motion-rejection algorithms and activity thresholds, previously validated by Gonzalez Utrilla et al. [21], which characterize specific signal morphologies for sedentary breathing versus high-intensity motion artifacts.

The findings from this in vitro validation have provided the essential safety and performance data required to progress to human trials. Consequently, a pediatric clinical investigation is currently underway, where the biosensor is being evaluated alongside full Polysomnography (PSG) in a clinical population [31]. This next phase aims to validate the device’s sensitivity and specificity in detecting central apnea in a real-world setting, integrating multimodal data (accelerometry, SpO_2_, and abdominal effort) to fully satisfy AASM diagnostic criteria. In those trials, the PneumoWave biosensor was successfully deployed alongside a wearable finger-worn pulse oximeter, validating the system’s ability to synchronize respiratory effort with oxygen saturation data in an ambulatory setting [31]. This established framework can be adapted for future pediatric home-monitoring protocols. Future pediatric clinical trials will also focus on optimizing and validating these algorithms for automated respiratory rate counting and distinguishing true apneic events from motion-induced noise.

## 5. Conclusions

This in vitro study demonstrated that the PneumoWave biosensor is a promising tool for detecting central sleep apnea in pediatrics. Its low cost, portability, and ability to continuously monitor and accurately record a wide range of respiratory rates and detect simulated apneic events merit progression into human volunteer trials.

## Figures and Tables

**Table 1:** Comparison of the PneumoWave biosensor with commercially available pediatric monitoring solutions.

Devices	Form	Regulatory Status	Data Accessibility	Primary Output	Target Use
Nanny Baby	Pad	Medical Device (Class IIB)	No	Acoustic/Visual Alarm	Apnea Alert
Snuza	Diaper clip	Medical Device (Class IIB)	No	Vibration/Audible Alarm	Apnea Alert
Nanit	Body band	Consumer Wellness	No	Video/App Notifications	Sleep Tracking
SISS Babycontrol^®^H	Wired Electrodes	Medical Device (Class IIB)	Statistics of the events	Alarm	Apnea Alert
PneumoWave	Adhesive Chest Sensor	Medical Device (Class 1)	Full Raw Waveform	Real-time Acceleration Signal	Clinical Diagnosis/Research

**Table 2:** Summary of in vitro manikin models and experimental conditions.

Manikin Model	Nominal Age	Lung Model	Respiratory Range	Tidal Volume/Pressure	Inspiration Time	Ventilator Model	3-Way Valve	References
PractiBaby	6-month	External	6–80 BPM	50 mL	0.3 s	LTV 1150	Yes	[22,23]
Resusci Baby	3-month	Internal	6–80 BPM (random)	3 cmH_2_O	0.3 s	SLE600	No	[24,25]
SimJunior	9-year	Internal	6–80 BPM	7 cmH_2_O	0.7 s	LTV 1150	No	[26,27]

## Data Availability

Data are available upon request by contacting the corresponding author.

## Abbreviations

The following abbreviations are used in this manuscript:

AASM	The American Academy of Sleep Medicine
BPM	Breaths per minute
CI	Confidence Interval
CSA	Central Sleep Apnea
CMV	Continuous Mechanical Ventilator
EEG	Electroencephalogram
EKG	Electrocardiogram
EMG	Electromyogram
ID	Inner Diameter
LoA	Limits of Agreement
MAVC	Manual Average Visual Count
UK	United Kingdom
